# Uniquely conserved immunosuppressive viral exoribonucleases

**DOI:** 10.18632/oncotarget.14548

**Published:** 2017-01-06

**Authors:** Bjoern Meyer, Hinh Ly

**Affiliations:** ^1^ Department of Veterinary and Biomedical Sciences, University of Minnesota, Twin Cities, MN, USA

**Keywords:** arenavirus, coronavirus, exoribonuclease, immune suppression, interferon

The interplay between viruses and host immune responses is critical to determine either a successful infection or resolution. Mammalian cells have developed different mechanisms to recognize invading pathogens *via* their pathogen-associated molecular patterns (PAMPs), which are often nucleic acid products (e.g., double-stranded RNAs or dsRNA) generated during viral genome replication [1]. To evade recognition of PAMPs by the cytosolic immune receptors (RIG-I or MDA5) and the subsequent activation of antiviral pathways, many viruses encode the so-called type I interferon (IFN) antagonists in their genomes. One such viral protein with enzymatic IFN antagonistic function, which was discovered by our laboratory [2], is the arenaviral nucleoprotein (NP). Arenaviruses are single-stranded negative-sense RNA viruses that are mainly rodent-borne zoonotic viruses that can cause severe and sometime fatal hemorrhagic fevers in humans. Lassa virus (LASV), for example, can cause up to half a million infections and 5,000 deaths in several countries in West Africa annually. We have recently shown that the 3’-5’ exoribonuclease function of the arenaviral NP, encoded at its C-terminal domain, is responsible for mediating type I IFN suppression [2]. Arenaviral NP exoribonuclease belongs to the DEDDh motif-containing exonuclease superfamily, members of which are often involved in RNA processing or repair pathways [3]. The main mode of action for the immunosuppressive function of NP, however, is thought to involve the destruction of immune stimulatory dsRNA generated during virus replication. Inactivation of the exoribonuclease activity results in a dramatic increase in IFN-induction through activation of the RIG-I and IRF3-dependent pathway, effectively inhibiting virus replication [4].

Besides arenaviruses, coronaviruses are the only other family of viruses known to encode the DEDDh exoribonuclease in the N-terminal domain of the non-structural protein 14 (nsp14) [5]. Coronaviruses are single-stranded positive-sense zoonotic RNA viruses, such as severe acute respiratory syndrome coronavirus (SARS-CoV) and Middle East respiratory coronavirus (MERS-CoV) that are responsible for severe and often fatal human infections. Until recently, nsp14 exoribonuclease activity has been exclusively attributed to its important proofreading role during viral RNA genome replication [6].

Becares and colleagues have recently investigated whether coronaviral nsp14 exoribonuclease can also inhibit type I IFN [7]. Recombinant viruses with mutations in one of the zinc-finger motifs located in the nsp14 exoribonuclease domain of the transmissible gastroenteritis virus (TGEV), a prototypic Alphacoronavirus, only mildly affect genome replication and transcription, but these mutations appear to result in less dsRNA accumulation in virus-infected cells. Interestingly, this reduction in the amount of dsRNA correlates with a decrease in the levels of type I IFN as well as a reduction in the mRNA levels of some representative interferon-stimulated genes (ISGs). The study’s authors conclude that coronaviral nsp14 exoribonuclease, similar to that of the arenaviral NP, has the potential to degrade immune stimulatory dsRNA during virus replication.

The enzymatic function of the viral DEDDh exoribonucleases plays important immune suppressive role in divergent virus families of arenaviruses and coronaviruses. New data suggest that in both cases these viral enzymatic proteins are involved in the destruction of immune stimulatory dsRNA, which normally triggers the type I IFN production. Interestingly, both nsp14 and NP do not only play important roles as type I IFN-antagonists but are also intimately involved in the viral genome replicative process. For example, nsp14 contributes to the high-fidelity replication of the relatively large genome of coronaviruses and possesses a secondary function with the viral genomic 5’ cap-modifying methyltransferase activity encoded in the protein’s C-terminal domain. While NP is a required component of the arenaviral replicase, its exact role during viral genome replication and transcription is not fully understood. Because the unique exoribonucleases of these diverse virus families play equally important roles during viral genome replication and transcription, and especially in mediating immune suppression, future research efforts can exploit this conserved DEDDh structural motif and its enzymatic function as a potential molecular target for the development of broad-spectrum antivirals against these deadly human viral pathogens.

## References

[1] Schlee M (2016). Nat Rev Immunol.

[2] Qi X (2010). Nature.

[3] Zuo Y (2001). Nucleic Acids Res.

[4] Meyer B (2016). J Virol.

[5] Minskaia E (2006).

[6] Sevajol M (2014). Virus Res.

[7] Becares M (2016). J Virol.

